# Post-concussion syndrome and concussion incidence improved in a pro rugby player following cervical spine rehab: case study and 6-year follow-up

**DOI:** 10.2217/cnc-2023-0004

**Published:** 2023-05-04

**Authors:** Seth Strauss, Douglas F Lightstone, Curtis Fedorchuk, Robert Pomahac, Paul A Oakley, Deed E Harrison

**Affiliations:** 1 Private Practice, 757 Long Point Road, Suite C, Mount Pleasant, SC 29464, USA; 2 Institute for Spinal Health & Performance, 460 Brannon Road, Suite 101, Cumming, GA 30041, USA; 3 CBP^®^ Non-Profit, 950 East Riverside Drive, Eagle, ID 83616, USA

**Keywords:** cervical lordosis, cervical spine alignment, cervical spine rehabilitation, Chiropractic BioPhysics^®^, concussion, coronal spinal posture, post-concussion syndrome, sagittal spinal posture, spine biomechanics, traumatic brain injury

## Abstract

**Aim:**

To report improvements in post-concussion syndrome and concussion incidence following cervical spinal alignment correction.

**Case presentation:**

A 27-year-old professional rugby player with 20 documented concussions presented with abnormal cervical spinal alignment and post-concussion syndrome. After 30 sessions of cervical rehabilitation, health outcomes improved. Post-treatment radiographs showed improved cervical lordosis from -13.5° to -37.4° (ideal is -42°) and right head translation from -22.7 to -11.3 mm (ideal is 0 mm). 2-year follow-up radiographs and 6-year follow-up health outcomes showed post-treatment improvements were maintained. The patient reported two documented concussions in the 6 years following treatment while maintaining the same lifestyle and professional rugby career.

**Conclusion:**

Correction of abnormal cervical spinal alignment may help athletes with post-concussion syndrome and reduce risk of concussion.

## Plain language summary

A 27-year-old male professional rugby player with a 6-year history of 20 documented concussions presented with severe neck and headache pain and disability. X-rays of his neck were taken to measure spinal alignment and posture. Also, neck and headache pain, disability and quality-of-life measures were assessed. After 30 treatment sessions of Chiropractic BioPhysics^®^ (CBP^®^) neck spinal alignment and posture rehabilitation, neck and headache pain and disability and quality-of-life improved. X-rays showed improved spinal alignment and posture in his neck. Follow-up x-rays 2 years after treatment showed that the improvements to his neck spinal alignment and posture were maintained. Follow-up assessment 6 years after treatment showed that the post-treatment improvements in in neck and headache pain and disability and quality of life were maintained. Additionally, the patient reported only two documented concussions in the 6 years following treatment maintaining the same lifestyle and competing as a professional rugby player at the same level, position and playing time. This is the first documented improvement in post-concussion syndrome and decreased concussion occurrence following correction of neck spinal alignment and posture. Correction of neck spinal alignment and posture allows the spine to function as a healthy shock absorber and increases its ability to decrease stress transferred to the brain from trauma responsible for concussions and may reduce the risk of concussions.

## Tweetable abstract

Improvements in post-concussion syndrome neck and headache pain and disability and concussion incidence in a professional rugby player following correction of abnormal spinal alignment and posture using Chiropractic BioPhysics^®^ Mirror Image^®^ structural spinal rehabilitation.

## Graphical abstract

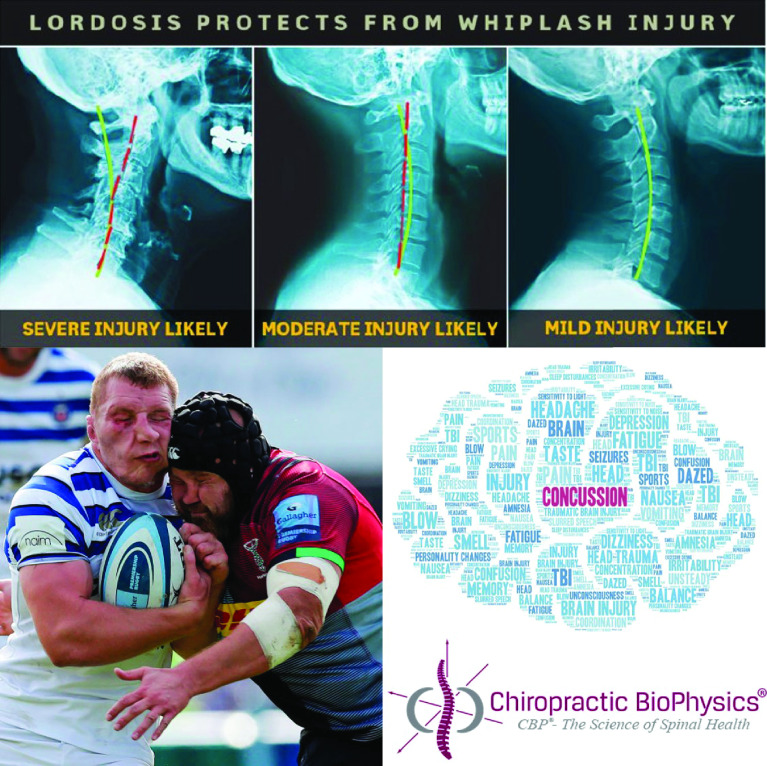

This paper is an expanded research article from an abstract presented at the Brain & Brain PET 2022 Conference in Glasgow, Scotland [1].

The number of concussion cases reported each year is rising, with 30–50 million new cases recorded globally and 3–4 million new cases reported in the USA [2]. A concussion is a traumatic brain injury either with or without loss of consciousness that occurs when an external biomechanical force to the head, neck or body is transmitted to the head and impairs brain function [2]. When symptoms and disabilities associated with concussion persist, it is known as post-concussion syndrome (PCS). Between 24 and 84% of mild traumatic brain injury patients develop PCS consisting of headaches, pain, dizziness, fatigue, sensory issues, sensitivity to light or sound, sleep disturbance, concentration problems and (or) other psychological, mental, emotional and (or) social difficulties. Concussion symptoms can become a chronic postinjury condition leading to a decreased quality of life [3].

Millions of youth and high school athletes sustain a sport related concussion each year. Interest and research into concussions have increased due to the likelihood for acute symptoms becoming chronic and the significant long-term neurologic sequalae that result both of which account for considerable morbidity [4]. Additionally, ‘multiple concussions appear to be a risk factor for cognitive impairment and mental health problems in some individuals’ [5]. Multiple and recurrent concussions is related to an increased incidence of unconsciousness, delayed recovery and an increased probability of the athlete being side-lined for the remainder of the season [6].

Concussion in rugby is common and a history of participation in rugby or concussions are associated with neurocognitive deficits. On average, rugby players recall more concussions than noncontact sport athletes. ‘Of the common injuries [in rugby], concussion has the highest match injury incidence, with the English Rugby Football Union reporting a rate of 15.8/1000 player-match-hours in 2015/2016’ [7]. Rugby players had a higher risk of concussion after 25 matches and those who sustained a concussion had a 60% increased risk of subsequent injury and subsequent injuries occur quicker after a concussion [7]. Former rugby players who reported at least one concussion experienced greater cognitive impairment than athletes who reported no history of concussions [8]. In addition, out of 22 documented PCS symptoms, headaches (57%) and neck pain (47%) were of the most common reported in South African collegiate male rugby players following a concussion [9].

As of 2020, no evidence-based guidelines exist for pharmaceutical therapy in the treatment of PCS. However, pharmaceuticals including over the counter anti-inflammatories and analgesics, amantadine, onabotulinumtoxinA (BOTOX^®^) and prescription antidepressants such as selective serotonin reuptake inhibitors and amitriptyline have been explored to address PCS symptoms [3]. Nutraceutical supplements have been investigated for potential benefits in PCS therapy. Antioxidants, B vitamins, omega-3 fatty acids, vitamin D, progesterone, melatonin, vitamin C and others have shown to provide protection and(or) aid in PCS recovery [3]. In select case reports, neurofeedback therapy, cognitive behavioral therapy, ocular rehabilitation, prescribed rest, physical therapy, osteopathic cranial manipulation, hyperbaric oxygen therapy and chiropractic manipulation have been associated with reduced or resolved PCS symptoms [3,10].

Since the biomechanics of concussions can involve complex acceleration/deceleration impacts and inertial loads applied to the cervical spine, skull, brain and neural tissue, it might be relevant to look at cervical spine alignment as a potential contributing or mitigating variable in the onset and sequelae of concussions. Studies have discussed the sagittal plane alignment of the cervical spine relative to cognitive dysfunction in whiplash injuries [11] and sports related cervical neurologic stinger injuries (spear-tacklers spine) [12]. However, there seem to be no studies investigating the effects of spinal alignment on concussion and(or) PCS. The objective of this study is to report on the effects of structural rehabilitation of the spine and posture using Chiropractic BioPhysics^®^ (CBP^®^) on symptoms, disabilities and quality of life associated with PCS of a 27-year-old professional rugby player with a history of repetitive concussions.

## Case presentation

An informed consent form for publication of this case was completed by the participant in this study. All procedures performed in this study were in accordance with the ethical standards of the 1964 Declaration of Helsinki and its later amendments or comparable ethical standards.

### Patient presentation

A 27-year-old Caucasian male with an athletic muscular build, a height of 193 cm, and a weight of 114 kg reported moderate-to-severe neck and headache pain he scored 7/10 on a scale of 0 (no pain) to 10 (maximum pain) resulting from a history of concussions. The patient was a starting forward professional rugby player for 6 years. Each year’s season consisted of approximately 30–40 rugby matches from June/July through May of the following year lasting 80 min per match.

### Patient history

The patient reported experiencing 20 documented diagnosed concussions in the previous 6 years, averaging one concussion per 11 matches every 16 weeks, as an elite professional rugby player who played for a national rugby team. On returning to play, the patient was required to pass 2 days of return to play protocol. Magnetic resonance imaging (MRI) of the head revealed normal signal from the brain, brainstem and cerebellum with no evidence of cerebral injury or extra-axial collection, no mass lesion, infarct or hydrocephalus, patent intracranial vessels and blood sensitivity. The patient stated that not all concussions resulted from head trauma, but those that did result from head knocks were sometimes followed by immediate amnesia which might last for differing amounts of time and approximately 1 day of irritability. The patient stated that he had been playing with the PCS symptoms documented. He reported previous treatments, including therapeutic exercises and physical therapy modality treatments from athletic trainers and physical therapists and traditional spinal manipulation from chiropractors, were not effective in relieving his post-concussion symptoms. These previous treatments were ongoing throughout his professional rugby career.

### Pretreatment exam findings

#### Patient outcome measures

##### Quadruple visual analog scale

The quadruple visual analog scale (QVAS) assesses four pain parameters (current, average, best and worst pain) on a visual analog scale of 0 (no pain) to 10 (maximum pain) [13]. An intensity score between 0 and 100 is calculated using current, average and worst pain scores. QVAS was administered at pretreatment, post-treatment and 6-year follow-up exams. QVAS changes of 1 are considered clinically meaningful [14]. The patient scored a pretreatment neck pain QVAS: current 10/10, best 2/10, worst 10/10 and average 7/10, providing an intensity score of 90/100 (high-intensity pain). The patient scored a pretreatment headache pain QVAS: current 10/10, best 0/10, worst 10/10 and average 7/10, providing an intensity score of 90/100 (high-intensity pain) (Table 1).

**Table 1.** Pretreatment, post-treatment and 6-year follow-up outcome assessment evaluations.

Outcome assessment	Pretreatment values	Post-treatment values	6-year follow-up values
**QVAS for neck pain (0–10, 0 = no pain, 10 = max pain; intensity score is 0–100, 0 = no intensity, 100 = max intensity)**			
Current	10/10	0/10	0/10
Best	2/10	0/10	0/10
Worst	10/10	5/10	4/10
Average	7/10	2/10	2/10
Intensity Score	90/100	23.3/100	20/100
**QVAS for headache (0–10, 0 = no pain, 10 = max pain; intensity score is 0–100, 0 = no intensity, 100 = max intensity)**			
Current	10/10	0/10	0/10
Best	0/10	0/10	0/10
Worst	10/10	5/10	4/10
Average	7/10	2/10	2/10
Intensity Score	90/100	23.3/100	20/100
**Neck Disability Index (total disability is 0–100%, 0% = no disability, 1–20% = minimal disability, 21–40% = moderate disability, 41–60% = severe disability, 61–80% = crippled, 81–100% = complete disability)**			
Pain Intensity	4/5	0/5	1/5
Personal Care	2/5	0/5	0/5
Lifting	5/5	0/5	0/5
Work	1/5	1/5	1/5
Headaches	3/5	1/5	1/5
Concentration	4/5	0/5	0/5
Sleeping	1/5	0/5	0/5
Driving	0/5	0/5	0/5
Reading	2/5	0/5	0/5
Recreation	4/5	0/5	0/5
Total disability	52/100	4/100	6/100
**Headache Disability Index (total disability is 0–100, 0 = no disability, 10–28 = mild disability, 30–48 = moderate disability, 50–70 = severe disability, 72–100 = complete disability)**			
Frequency	>1 HA/week	1 HA/month	1 HA/month
Intensity	Moderate	Mild	Mild
Functional disability	22/52	2/52	4/52
Emotional disability	20/48	6/48	4/48
Total disability	44/100	8/100	8/100
**SF-36 HRQOL Questionnaire (0–100, 0 = worst quality of life, 100 = best quality of life)**			
PF	45/100	95/100	95/100
BP	22.5/100	90/100	90/100
RP	0/100	100/100	100/100
RE	100/100	100/100	100/100
MH	92/100	92/100	92/100
SF	100/100	100/100	100/100
VIT	85/100	100/100	100/100
GH	85/100	100/100	90/100
ΔH	50/100	100/100	75/100

Shows comparisons of pretreatment, post-treatment and 6-year follow-up values for PCS symptoms including neck and headache pain and disability values and health related quality of life values.

PCS: Post-concussion syndrome; QVAS: Quadruple visual analog scale; SF-36 HRQOL: Short-form 36-question health-related quality of life.

##### Neck Disability Index

The Neck Disability Index (NDI) is used to assess the day-to-day impact of neck pain and to quantify and qualify disability due to neck pain [15]. NDI was administered at pretreatment, post-treatment and 6-year follow-up exams. NDI changes of 21% are considered clinically meaningful [16]. On pretreatment NDI, the patient scored a 52% indicating severe disability in daily activities due to neck pain (Table 1).

##### Headache Disability Index

The Headache Disability Index (HDI) is used to assess the day-to-day impact of headache pain and to and to quantify and qualify disability due to headaches [17]. HDI was administered at pretreatment, post-treatment and 6-year follow-up exams. HDI changes of 29 points are considered clinically meaningful [18]. On pretreatment HDI, the patient reported a headache frequency of more than one headache per week and scored a 42 indicating moderate disability in daily activities due to headaches (Table 1).

##### Short-form 36 health related quality of life questionnaire

The Short Form-36 (SF-36) is a 36-question survey that provides scaled scores for health-related quality of life (HRQOL) in nine different domains from 0 (lowest HRQOL) to 100 (highest HRQOL) [19]. SF-36 was administered at pretreatment, post-treatment and 6-year follow-up exams. SF-36 changes of 5 points are considered clinically meaningful [20]. Pretreatment SF-36 showed: Physical Functioning (PF) was 45, Bodily Pain (BP) was 22.5, Role limitations due to physical health problems (RP) was 0, role limitations due to personal or emotional problems (RE) was 100, mental health (MH) was 92, social functioning (SF) was 100, energy/fatigue or vitality (VIT) was 85, general health (GH) was 85 and change in health status (ΔH) was 50 (Table 1).

##### Radiographic analysis

A valid and reliable way to evaluate abnormalities, aberrant posture and spinal alignment is by radiographic analysis. Spinal abnormalities include ‘rotations and translations of the head, rib cage and pelvis from a normal position in a 3D coordinate system’ [21–23]. Measurements from the spinal radiograph analyses are used to determine the best strategies for structural spinal rehabilitation [22,23].

A neutral lateral cervical (NLC) radiograph of the patient was examined per the Harrison Posterior Tangent Method [24,25] in accordance with the Harrison Spinal Model [26–28]. On lateral spinal radiographs, juxtaposed posterior tangent lines of the vertebrae provide intervertebral and spinal region angles. C2–C7 posterior tangents provide the absolute rotation angle cervical lordosis measurement (ARA C2–C7). Horizontal displacements of a spinal region are measured at a superior vertebral landmark from a vertical sagittal axis from an inferior vertebral landmark. These spinal measurements are compared with valid, normal, ideal values [21–28].

A shorthand identifies spine alignment. Positive or negative measurements indicate the rotation (R) around or translation (T) in the three axes (x, y or z) of the head (H), thorax (T) or pelvis (P) or any specific vertebra.

**Figure 1.**
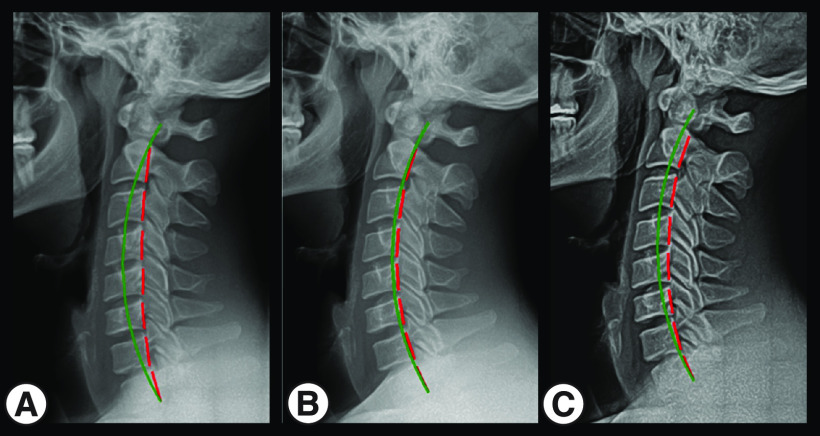
(A) Pretreatment, (B) post-treatment and (C) 2-year follow-up neutral lateral cervical radiographs.

**Figure 2.**
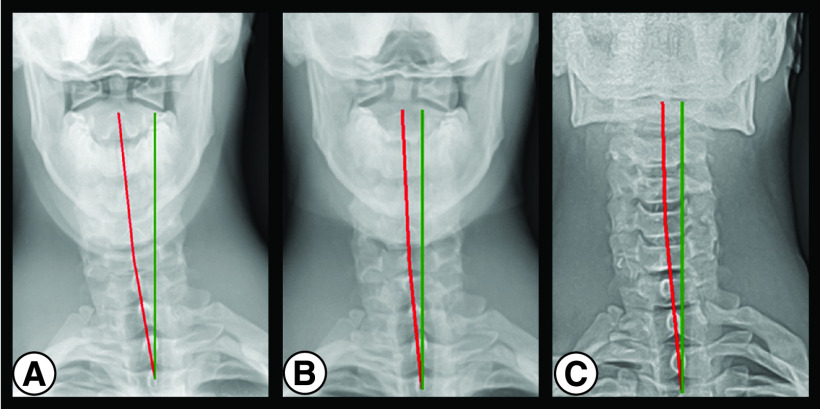
(A) Pretreatment, (B) post-treatment, and (C) 2-year follow-up anterior-to-posterior lower cervical radiographs.

Pretreatment NLC radiograph (Figure 1A) shows ARA C2–C7 measuring -13.5° (average value is -34°; ideal is -42°) [26–28]. Pretreatment anteroposterior lower cervical (APLC) radiograph (Figure 2A) shows Tx C2–C3 measuring -22.7 mm (ideal is 0 mm) (Table 2) [21–23].

**Table 2.** Pretreatment, post-treatment and 2-year follow-up cervical spine radiograph measurements.

Cervical spine measurements	Normal values	Pretreatment radiograph values	Post-treatment radiograph values	1-year follow-up radiograph values
ARA C2-C7	-42.0°	-13.5°	-37.4°	-36.7°
Tx C2-T3	0.0 mm	-22.7 mm	-11.3 mm	-11.7 mm

Shows comparisons of normal, pretreatment, post-treatment and 2-year follow-up radiograph values of cervical sagittal curvature (ARA) of designated vertebrae and cervical lateral translation (Tx) of designated vertebrae.

### Treatment

Over a period of 16 days, the patient underwent 30 sessions of CBP^®^ Mirror Image^®^ spinal adjustments, exercises and traction in a private practice under the care of a doctor of chiropractic with advanced training in CBP^®^ which includes 190 hours of combined lecture and practical instruction as well as a case presentation of clinical success in practice subject to peer review. To correct spinal alignment and posture, Mirror Image^®^ structural spinal rehabilitation requires placing the patient into the corrected or overcorrected alignment and postural position [21,23].

#### Mirror Image^®^ adjustments

Mirror Image^®^ spinal adjustments are given with the patient positioned in their corrected or overcorrected spinal alignment and posture and stimulate mechanoreceptors and proprioceptors [21,23,29] to train the patient’s CNS to change [23,29].

Mirror Image^®^ adjustments were administered using an OMNI elevation drop table and an Impulse^®^ adjusting instrument (Impulse^®^ Adjusting Instrument, Neuromechanical Innovations, AZ, USA). Mirror Image^®^ adjustments consisted of left head translation and cervical extension.

#### Mirror Image^®^ exercises

Mirror Image^®^ exercises strengthen and lengthen respective muscles that have maladapted to abnormal spinal alignment and posture [21,23]. The patient was trained to perform the exercises and was observed while doing so. Left head translation and cervical extension were performed with cycles of contraction and relaxation for a total of 8–10 minutes. The patient was directed to contract for 15 seconds in the Mirror Image^®^ posture and then relax for 5 seconds.

#### Mirror Image^®^ traction

Mirror Image^®^ traction creates long-term, restorative plastic deformation of the spine [22,23,29] by loading spinal connective tissue and initiating muscle creep resulting in long-lasting correction [22,23,29]. Mirror Image^®^ spinal traction consisted of left head translation and cervical extension using Denneroll^TM^ Spinal Orthotics (Denneroll^TM^ Spinal Orthotics, New South Wales, Australia) and CBP^®^ Mirror Image^®^ Blocks (CBP^®^ Seminars, Inc., ID, USA) [29]. The patient started with 8 minutes and worked up to 15 minutes of traction per setup with each session.

### Post-treatment exam findings

Thirty treatment sessions were administered over 16 days. Post-treatment exam was performed 24 hours following the last treatment session and showed improved PCS neck and headache pain, disability and HRQOL. Post-treatment neck pain QVAS revealed improved occasional neck pain: current 0/10, best 0/10, worst 5/10, average 2/10, providing an intensity score of 23.3/100 (low-intensity pain) (Table 1). Post-treatment headache pain QVAS revealed improved infrequent headache pain: current 0/10, worst 5/10, average 2/10, providing an intensity score of 23.3/100 (low-intensity pain) (Table 1). Post-treatment NDI improved to 4% indicating minimal disability due to neck pain (Table 1). Post-treatment HDI improved to 8 indicating minimal disability due to headache pain (Table 1). Post-treatment SF-36 improved in: PF to 95, BP to 90, RP to 100, VIT to 100, GH to 100 and ΔH to 100 (Table 1).

The post-treatment NLC radiograph (Figure 1B) shows improved ARA C2–C7 to -37.4° (ideal is -42.0°). Post-treatment APLC radiograph (Figure 2B) shows improved Tx C2–T3 to -11.3 mm (ideal is 0 mm) (Table 2).

### 2-year follow-up radiograph findings

The patient did not receive corrective spinal rehabilitation following his 30 visits. In the 2 years following treatment, the patient continued to compete as an elite professional rugby player. The patient was a starting forward for a professional rugby team competing in approximately 30–40 rugby matches from June/July through May of the following year in matches lasting 80 min. NLC and APLC radiographs showed that post-treatment improvements in ARA C2-C7 and Tx C2-T3 were maintained at 2-year follow-up (Figure 1C & 2C & Table 2).

### 6-year follow-up exam findings

Neck and headache pain QVAS, NDI, HDI and SF-36 showed that post-treatment improvements in neck and headache pain and disability and HRQOL were maintained at 6-year follow-up (Table 1).

The patient reported that since his re-exam 6 years prior, he had sustained two documented diagnosed concussions while maintaining the same lifestyle and competing in the same sport at the same level, position and playing time. The patient stated that both his spinal and general health had significantly improved. He claimed to have occasional, minimal pain but otherwise to be enjoying life and able to carry out activities of daily living and extended activities without restrictions.

## Discussion

This case shows maintained improvements in PCS symptoms and significant reduction in concussion incidence of a professional rugby player following corrective spinal rehabilitation of cervical spine.

### Cervical spine & health

A lordotic curvature exists in a normal sagittal cervical spine [26–28]. Spinal cord and nerve strain, pain, impairment and diminished health and HRQOL are associated with cervical hypolordosis and kyphosis [21–23,28,30] due to long-term adverse neural tension and deformed tissue [30,31] caused by the abnormal spinal alignment and posture [21–23,30,31]. This mechanical strain causes intervertebral disc and facet joint degeneration [32]. Increased spine loading caused by poor spinal alignment has deleterious effects on healthy tissue development and healing [21,22,27]. Prolonged abnormal posture leads to neural tension, increased intramedullary and cerebrospinal fluid pressure and diminished afferent and efferent nerve function [31,33–35].

In studies, structural rehabilitation of the cervical spine is associated with improved neurological, muscular and skeletal conditions including cervical spondylotic radiculopathy [34], neck pain [35,36], segmental spinal motion [37], discogenic cervical radiculopathy [35], cervicocephalic kinesthetic sensibility [36], central conduction time and neuroplasticity [31,33,35], as well as visceral type conditions like dizziness and cervicogenic headaches [36,38].

### Cervical spine, concussion & post-concussion syndrome

According to Wolff’s Law, reduced cervical curvature causes substantial increased vertebral pressure, resulting in spinal compression, arthritis and degeneration [32]. These skeletal changes create biomechanical instability requiring the connective tissues that support the neck to work harder resulting in muscle and soft tissue weakness and injury, per Davis’s Law [39,40]. Furthermore, the vasculature within the spine will be subjected to prolonged abnormal stresses and strains [41–43].

Vertebral artery hemodynamics [44] and cerebral blood flow (CBF) [45] has been linked to cervical lordosis. Improved of cervical curvature increases cerebral blood flow, which is consistent with the notion that spinal biomechanics affects physiology [45]. Data shows as cervical lordosis improves, so too does CBF [45]. Arteries lose their elasticity and stiffen as a viscoelastic response to increased tension from cervical hypolordosis or kyphosis [46,47].

Since the cerebral arteries supply blood to the brain [48], cervicogenic headache may be connected to altered cerebral vascularization in addition to its relation to abnormal cervical spine structures [38,49]. *Wang et. al.* identified that athletes with concussions showed a reduced CBF compared with controls who demonstrated no change [50]. Increased CBF following sports related concussions decreased the extent of symptoms ‘suggesting a potential prognostic indication for CBF as a biomarker’ [51].

CBP^®^ spinal rehabilitation is an effective, conservative method of correcting abnormal spinal alignment and posture [21–23,29,30]. In this case study, restoring cervical spinal alignment and posture was associated with improvement in PCS symptoms, pain, disabilities and concussion frequency. It stands to reason that improved cervical spine and posture alignment decreased unhealthy biomechanics affecting neuromuscular tissues [32,52]. It is possible that correction of the cervical lordotic curvature and improvement in our patient’s lateral head translation were associated with an increased ability of the cervical spine to attenuate loads transferred to the brain tissue via the impact responsible for concussions and PCS. In this manner, spinal curvatures are the primary shock absorbers [53]. Thus, the reduction in the number of concussions from 20 to 2 over the same time period (6 years before corrective care versus 6 years following spine correction) in our patient might be related to improved biomechanics of the cervical lordosis and the neutral spine. At the very least, this warrants further biomechanical investigation.

### Study limitations

As is true for case reports, this study has limitations in being able to draw conclusions about correlation, causation or applying the findings to broader spectrum of varying demographics. While the detrimental health effects of reduced cervical curvature are evident [22,28,30,33–38,45], there are no studies on the effects of cervical curvature as it relates to concussion and PCS. Clinical trials involving patients with concussions and spinal alignment, structural spinal rehabilitation, medical and surgical management and control groups with long-term follow-ups are needed. Investigating the relationship between cervical spinal alignment and vascular, neurological and other system changes would be beneficial for considerations for spine, concussion and PCS management.

## Conclusion

This case study contributes to the growing body of research showing that structural rehabilitation of the spine and posture using CBP^®^ can be an effective long-term, conservative treatment without the use of pharmaceuticals or surgery for neurological, muscular and skeletal conditions as well as non-musculoskeletal conditions including pain, disability and reduced HRQOL. This study is the first to indicate that improvement in symptoms, disabilities and HRQOL from PCS as well as a decreased frequency of concussions may be associated with correction of abnormal cervical spinal alignment. Structural spinal rehabilitation could be utilized to avoid degenerative spine diseases and their consequences. Further research is necessary to evaluate the clinical significance of this, including rates and susceptibility for PCS, concussions and traumatic brain injury.

**Summary points**

- Concussion in rugby is common and a history of participation in rugby or concussions are associated with neurocognitive deficits.
- Since the biomechanics of concussions can involve complex acceleration/deceleration impacts and inertial loads applied to the cervical spine, skull, brain, and neural tissue, it might be relevant to look at cervical spine alignment as a potential contributing or mitigating variable in the onset and sequelae of concussions.
- This case study reports on a 27-year-old professional rugby player with 20 documented concussions over 6 years and post-concussion syndrome who underwent 30 treatment sessions of cervical rehabilitation using Chiropractic BioPhysics^®^.
- A 27-year-old professional rugby player with a history of concussions and PCS reported improvements in concussion incidence and post-concussion syndrome symptoms including neck and headache pain and disability and quality of life following correction of sagittal and frontal cervical spinal alignment.
- A 2-year follow-up examination of a 27-year-old professional rugby player with a history of concussions and PCS showed that post-treatment improvements in sagittal and frontal cervical spinal alignment were maintained.
- A 6-year follow-up examination of a 27-year-old professional rugby player with a history of concussions and PCS showed that post-treatment improvements in the neck and headache and disability and quality of life were maintained.
- A 27-year-old professional rugby player with a history of 20 concussions over 6 years and PCS reported that in the 6 years following correction of sagittal and frontal cervical spinal alignment he had sustained only two documented diagnosed concussions while maintaining the same lifestyle and competing as a professional rugby player at the same level, position and playing time.
- Correction of sagittal and frontal cervical spinal alignment allows the cervical spine to function as a primary shock absorber and increases its ability to attenuate loads transferred to the brain tissue via impacts responsible for concussions which may reduce the risk of concussions.
